# Quality of YouTube Videos on Laparoscopic Pyeloplasty in Children: An Independent Assessment by Two Pediatric Surgeons

**DOI:** 10.7759/cureus.17085

**Published:** 2021-08-11

**Authors:** Sachit Anand, Bhushanrao Jadhav, Gursev Sandlas

**Affiliations:** 1 Pediatric Surgery, All India Institute of Medical Sciences, New Delhi, IND; 2 Pediatric Surgery, Noah's Ark Children's Hospital, Cardiff, GBR; 3 Pediatric Surgery, Kokilaben Dhirubhai Ambani Hospital and Medical Research Institute, Mumbai, IND

**Keywords:** e-learning, operative video, youtube, internet, laparoscopic pyeloplasty, children

## Abstract

Background

YouTube (YT) is the most common video platform accessed by surgical trainees for the preparation of surgery. However, the quality of the YT videos has been questioned time and again. This study was performed to comprehensively assess the quality of the available YT videos on pediatric laparoscopic pyeloplasty (LP).

Materials and Methods

The term “laparoscopic pyeloplasty in children” was searched in YT on June 3, 2021, and ten most-viewed videos on LP were included. The percentage video power index (%VPI), the Journal of American Medical Association (JAMA) benchmark criteria, and the laparoscopic surgery video educational guidelines (LAP-VEGaS) video assessment tool were used to assess the video popularity, the quality of medical information, and the overall quality of the included videos respectively. Videos were defined as acceptable (score of 11 or more) or poor quality (score <11) based on LAP-VEGaS scores. The inter-observer agreement, in terms of the LAP-VEGaS scoring, was observed among two surgeons using the kappa statistics.

Results

The median values of the %VPI and JAMA scores of the included YT videos were 68.1 (range 0-13570) and 2 (range 1-2) respectively. The median LAP-VEGaS score of these videos was 6.75 (range 2-16.5) with only two videos having acceptable quality. The quality of these videos was poor in 7/9 domains of the LAP-VEGaS tool. A moderate inter-observer agreement (kappa=0.542) was observed in terms of the LAP-VEGaS scores assigned to the videos (p<0.0001).

Conclusion

A comprehensive assessment of the ten most-viewed YT videos on pediatric LP revealed poor overall quality. The included videos depicted sub-optimal presentation of the medical information and weak conformity to the LAP-VEGaS guidelines.

## Introduction

Laparoscopic pyeloplasty (LP) is one of the common minimally invasive procedures performed in children [1]. Recent studies have demonstrated the safety and efficacy of LP in infants as well [2]. Due to the recent popularity of this approach, pediatric LP has been incorporated in the training programs of various centers; senior trainees are expected to perform LP at the end of their multimodal learning program [3]. It has been demonstrated that a combination of hands-on experience on endo-trainer and watching operative videos provides an ideal training of the laparoscopic procedures [4,5].

Over the past few decades, e-learning has emerged out to be the new method of learning among medical undergraduates and graduates [6]. In fact, residents and surgical trainees often watch operative videos on various video platforms as a part of their procedural preparation [7]. YouTube (YT) is trainees' most common video platform to access operative videos [8]. It is well-known that the freely accessible content of YT has made it popular among the trainees, however, the quality and reliability of the information provided by the YT videos is really questionable [9,10].

By utilizing three independent video assessment tools, the current study aims to assess the quality of operative videos regarding LP on YT. Percentage video power index (%VPI), the Journal of American Medical Association (JAMA) benchmark criteria, and the laparoscopic surgery video educational guidelines (LAP-VEGaS) video assessment tool were used to assess the video popularity, the quality of medical information, and the overall quality of the included videos respectively. We hypothesize that the quality of these YT videos is sub-optimal and poor. We also intend to evaluate the inter-observer agreement on LAP-VEGaS scoring among two surgeons with different operative experiences.

## Materials and methods

One of the authors (SA) utilized the ‘advanced search’ feature of the Google search engine on June 3, 2021, to identify the total number of YT videos on pediatric laparoscopic pyeloplasty. After entering the search term as “laparoscopic pyeloplasty in children” (minus ‘robot’) in the ‘all these words’ menu and entering the domain as youtube.com, the total number of YT videos were identified. Further, on the same day, an independent video search was conducted by two authors (SA and BJ) to screen the operative videos regarding LP in children on YT. The videos were filtered as per their view counts and ten most-viewed videos were selected. Seminars, lectures, webinars, and commercial advertisements were excluded. Videos depicting open pyeloplasty were also excluded.

Video characteristics such as the information about the surgeon (name and country), type of the operative approach (retroperitoneal or transperitoneal), operative side (left or right), year of video upload, the duration of the video, view count, like count, and dislike count were recorded. An estimate of the video popularity, the video power index (VPI), was calculated as done in the previous studies [11]. The Journal of American Medical Association (JAMA) benchmark criteria was used to evaluate the quality of the health-related information in the included videos [12]. Each video was evaluated under four domains- authorship, attribution, disclosure, and currency. The grading of each domain was done from 0-1, yielding minimum and maximum scores of 0 and four respectively. 

Two authors (BJ and GS), with five years and more than ten years of experience in pediatric laparoscopic surgery respectively, assessed the quality of the operative videos using the LAP-VEGaS tool [5]. Developed in accordance with the Laparoscopic surgery video educational guidelines (LAP-VEGaS), this tool has nine domains of assessment. Each domain was graded from 0-2, with minimum and maximum scores of 0 and 18 respectively. Based on the assigned scores, the videos were defined as poor quality (score <11) or acceptable quality (score of 11 or more).

This cross-sectional study involved the analysis of information already available on an open video platform, i.e. YouTube. A clearance from Institutional Review Board was not required as no patient contact was established throughout the course of the study. Data entry was done in Microsoft Excel (version 15.24) spreadsheets and analyses were performed using StataCorp. 2011. Stata Statistical Software: Release 12. College Station, TX: StataCorp LP. Data were expressed as numbers, proportion, median, and ranges. Wilcoxon rank-sum test was used to compare the median values of percentage VPI (%VPI) and JAMA scores among the acceptable quality (group A) and poor quality (group B) videos. The inter-observer agreement for LAP-VEGaS scores was adjudged using the kappa statistics [13]. Based on the value of kappa, the level of agreement was defined as almost perfect (0.81-1.00), substantial (0.61-0.80), moderate (0.41-0.60), fair (0.21-0.40), slight (0.00-0.20), and poor (<0.00). A p-value of <0.05 was considered to be statistically significant.

## Results

A total of 125 videos were available on YT regarding pediatric LP. Of these, thirteen most-viewed videos were screened to select ten of them. Two videos were excluded because one was a webinar and the other was a lecture. The third excluded video depicted LP performed by the senior author (GS) of the study. The surgeon’s information was present in all of the included videos. The majority (6/10; 60%) of the videos were uploaded by surgeons from India. The countries of origin of the remaining videos were France (n=1), the United States of America (n=1), Russia (n=1), and Brazil (n=1). Table 1 depicts the baseline characteristics of the included videos.

**Table 1:** Baseline characteristics of the included videos USA: The United States of America, TP: transperitoneal, RP: retroperitoneal *Details not available †Of all the children undergoing laparoscopic pyeloplasty via the transperitoneal approach, access to the renal pelvis in this child was established via the retrocolic route. In the rest of the cases, the access was via the transmesenteric route.

S N	Country	Year	Duration (minutes)	View count	Likes	Dislikes	Operative side	Operative approach
1	India	2014	5.32	454168	741	207	Left	TP
2	India	2015	6.90	13734	28	7	Right	TP
3	India	2017	5.05	5862	29	4	Right	TP
4	France	2013	10.80	5306	20	0	Right	RP
5	USA	2010	6.60	2892	4	1	Left	TP
6	India	2014	4.20	1929	4	4	Right	TP
7	Russia	2015	14.45	1629	14	1	Left	TP^†^
8	Brazil	2012	6.10	1528	2	0	*	*
9	India	2014	12.65	1316	0	1	Left	TP
10	India	2016	4.25	985	1	0	Left	TP

The ten most-viewed videos regarding pediatric LP on YouTube were uploaded between 2010-2017. The median duration of the videos was 6.35 (range= 4.20-14.45) minutes. The median view count, like count, and dislike count were 2411 (range= 985-454168), 9 (range 0-741), and 1 (range 0-7). Five out of ten videos depicted left-sided pathology. The transperitoneal approach was used in the majority (8/10; 50%) of the children. The operative side and operative approach were not mentioned/appreciable in one of the videos.

Table 2 depicts the scores assigned to each video utilizing the three assessment tools. The median %VPI and JAMA scores of the included YT videos were 68.1 (range 0-13570) and 2 (range 1-2) respectively. The median LAP-VEGaS score of these videos was 6.75 (range 2-16.5). Only two videos (20%) had an acceptable quality and belonged to group A. No significant differences were observed among the two groups of videos in terms of the %VPI (p=0.79) and JAMA score (p=0.22). A moderate inter-observer agreement (kappa=0.542) was noticed in terms of the LAP-VEGaS scores assigned to the videos (p<0.0001).

**Table 2:** Scores assigned to all the included videos using the three assessment tools VPI: Video Power Index, JAMA: Journal of American Medical Association benchmark criteria. *arranged in order of decreasing video count Q1-Q9: Nine domains of the laparoscopic surgery video educational guidelines (LAP-VEGaS) assessment tool. The average scores (of both the observers) assigned to each video in the respective domains are depicted in the table.

SN*	%VPI	JAMA	Q1	Q2	Q3	Q4	Q5	Q6	Q7	Q8	Q9	Total
1	13570	2	2	1	0	0.5	1	1	1	2	1	9.5
2	543.4	2	1.5	0	0	1.5	1.5	0.5	0	2	1	8
3	336.7	1	0	0	0	1	1.5	0	0	0	1	3.5
4	178.4	2	2	2	2	2	2	1	1.5	2	2	16.5
5	60.4	2	2	1	2	2	1	1.5	2	0	2	13.5
6	36.0	1	0	0	0	1	1	0	0.5	0	0	2.5
7	75.8	1	0	0	0	1	1	0	0	1	0	3
8	46.6	2	0.5	0.5	0	1	1.5	0	0	2	0	5.5
9	0	1	1	0	0	1	0	0	0	0	0	2
10	59.2	2	1.5	2	1	1.5	1.5	0	0.5	1	0	9

**Figure 1:**
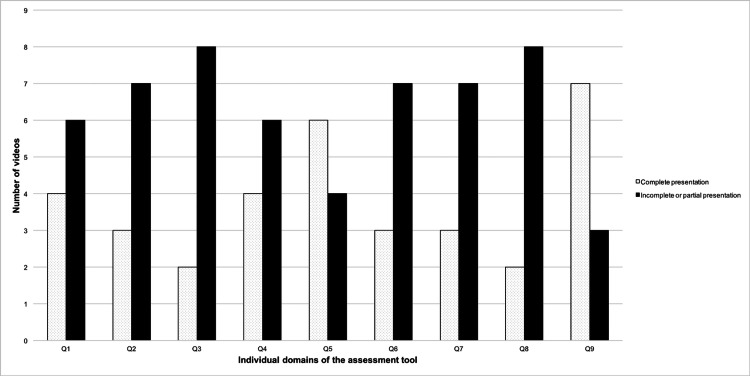
Distribution of the scores assigned to the YouTube videos in the individual domains of the LAP-VEGaS tool Q1-Q9: Nine domains of the laparoscopic surgery video educational guidelines (LAP-VEGaS) assessment tool

Figure 1 depicts the distribution of the scores assigned to the YT videos in the individual domains of the LAP-VEGaS tool. The quality of the included videos was poor in seven out of nine domains. In these domains, the information presented by the majority of the videos was either absent or partially presented. Eight out of ten videos either lacked an audio (or written) commentary (item 8) or had an incomplete description about the patient position, access ports, and surgical team (item 3). Also, 70% of the videos failed to demonstrate the formal case presentation (item 2), relevant outcome data (item 6), and additional graphic aids (item 7). In addition, 60% of the videos failed to depict the author’s details (item 1) or didn’t describe the procedure in a step-by-step fashion (item 4). On the other hand, more than 50% of the videos clearly demonstrated the intraoperative findings (item 5) with an appropriate image quality (item 9).

## Discussion

The operative exposure of the residents and trainees varies considerably among different surgical subspecialties. In fact, due to the worldwide variation in weekly working hours, inconsistent surgical exposure is observed among trainees of similar subspecialty but working in different centers of the world [4]. These variations ultimately affect the learning curve of the trainees. To overcome this, the residents often resort to surgical videos on different video platforms.

YT is the most commonly used video platform by trainees from different surgical sub-specialties [4,7]. Although there are numerous advantages of watching procedural videos on YT, previous studies have demonstrated that the quality of these videos is highly variable [7]. de’ Angelis et al. have demonstrated the poor quality of available YT videos on laparoscopic appendectomy [10]. Similarly, Rodriguez et al. have questioned the technical aspects of the YT videos regarding laparoscopic cholecystectomy [9].

In this study, a comprehensive quality assessment of the YT videos on pediatric LP was performed. %VPI was used as the tool for the assessment of video popularity. The reason for using %VPI rather than the individual parameters (views, likes, dislikes) was to avoid any bias due to the year of uploading the video [11,14]. A consistent observation was seen in our study; where the videos uploaded in the years 2014, 2015, and 2017 had the maximum %VPI rather than the videos which were uploaded before 2014. The JAMA benchmark criteria were used to assess the quality and reliability of health-related information in these videos. Silberg et al. had suggested that the quality of the medical information in web-based sources is not optimal if three out of four domains of the JAMA criteria are not fulfilled [12]. As all the YT videos had JAMA scores of ≤2, a sub-optimal quality was declared in terms of the medical information disseminated by them.

LAP-VEGaS was the third tool that was used to assess the overall quality of the operative videos on minimally-invasive surgery (MIS) in the current study. Weak conformity to the LAP-VEGaS guidelines was observed among the included videos. Only two videos had an acceptable quality (score of 11 or more). Apart from the two domains (items 5 and 9), the domain information was incompletely presented (or not presented) by the majority of the videos. Consistent with the findings of the previous studies [5], a moderate and statistically significant agreement existed among the observers in terms of the LAP-VEGaS scoring. No significant differences were observed among the videos of group A versus group B in terms of the %VPI or JAMA, suggesting that the overall quality of the minimally-invasive procedure (depicted by LAP-VEGaS) is an independent variable and has no relation with video popularity or medical information contained within the video.

Although Haslam et al. [15] have demonstrated similar findings of low conformity to the LAP-VEGaS guidelines among the YT videos on pediatric robotic pyeloplasty, however, there is a paucity of published literature on the quality of YT videos on LP in children. To our best knowledge, ours is the first study to comprehensively assess the quality of surgical videos on LP in children.

The present study has few limitations. First, we have included ten most-viewed operative videos on pediatric LP from one video platform only. The sample size of the study is small. Also, a comparison with other video platforms and video libraries of the surgical societies will provide insightful information. Second, only videos regarding one surgical procedure were included in our study. Therefore, the available YT videos on other pediatric minimally-invasive procedures need to be scrutinized before any definite conclusions are drawn. Finally, for the quality assessment, the videos were filtered as per the view counts. As depicted by the lack of correlation between high LAP-VEGaS scores and %VPI, the criterion to filter the videos based on their view counts is not ideal. Hence, further studies with well-structured screening criteria need to be conducted for an optimal assessment of the video quality.

## Conclusions

A comprehensive assessment of the ten most-viewed YT videos on pediatric LP revealed poor overall quality. The included videos depicted a sub-optimal presentation of the medical information (JAMA benchmark criteria) and weak conformity to the LAP-VEGaS guidelines. Therefore, strict viewer discretion is advised to the surgical trainees while watching the operative videos on YT till compliance with these quality assessment tools is significantly improved.
